# Akt activation by human T-cell leukemia virus tax oncoprotein

**DOI:** 10.1186/1742-4690-12-S1-P54

**Published:** 2015-08-28

**Authors:** Mathew Cherian, Hicham Baydoun, Jacob Al-Saleem, Mamuka Kvaratskhelia, Patrick Green, Lee Ratner

**Affiliations:** 1 Division of Oncology, Washington University, St Louis, MO, USA; 2 Center for Retrovirus Research, The Ohio State University, Columbus, OH, USA

## 

Human T-Cell Leukemia Virus type 1 (HTLV-1), the etiological agent of Adult T-Cell Leukemia, expresses the viral oncoprotein Tax1. In contrast, HTLV-2, which expresses Tax2, is non-leukemogenic. One difference between these homologous proteins is the presence of a C-terminal PDZ (post synaptic density protein) domain binding motif (PBM), previously reported to be important for non-canonical nuclear factor kappa B (NFkappaB) activation. In contrast, the current study finds no defect in non-canonical NFkappaB activity by deletion of the Tax1 PBM. Instead, Tax1 PBM was found to be important for Akt (Protein Kinase B) activation. Tax1 attenuated the effects of negative regulatory phosphatases of the PI3K-Akt-mTOR pathway, PTEN (Phosphatase and Tensin homologue) and PHLPP (PH domain and leucine rich repeat protein phosphatase). Tax1 competes with PTEN for binding to PDZ protein DLG-1 (Drosophila disc large tumor suppressor), unlike a PBM deletion mutant of Tax1. Forced membrane expression of PTEN or PHLPP, by fusion to a myristolyation acceptor motif, overcame the effects of Tax1, as measured by levels of Akt phosphorylation at T308 and S473, and rates of Akt dephosphorylation. The current findings suggest that Akt activation may explain the differences in transforming activity of HTLV-1 and -2. Moreover, these findings suggest a new approach to therapeutics for HTLV-1 lymphoproliferative disease. Supported by NIH grants CA94056, CA1730, CA63413, Lymphoma Leukemia Society grant 6067-10, American Society for Hematology fellowship and NIH T32 grant HL07088, and Lymphoma Research Foundation grant 307181203.

